# Fermented Lettuce Extract Containing Nitric Oxide Metabolites Attenuates Inflammatory Parameters in Model Mice and in Human Fibroblast-Like Synoviocytes

**DOI:** 10.3390/nu15051106

**Published:** 2023-02-23

**Authors:** Jisu Park, Ji Hyeon Ryu, Bo-Young Kim, Hyun Soo Chun, Min Sun Kim, Yong-Il Shin

**Affiliations:** 1 Research Institute for Convergence of Biomedical Science and Technology, Pusan National University Yangsan Hospital, Yangsan 50612, Republic of Korea; 2 HumanEnos LLC., Wanju 55347, Republic of Korea; 3 Center for Nitric Oxide Metabolite, Wonkwang University, Iksan 54538, Republic of Korea; 4 Department of Rehabilitation Medicine, School of Medicine, Pusan National University, Yangsan 50612, Republic of Korea

**Keywords:** rheumatoid arthritis, collagen-induced arthritis, fibroblast-like synoviocytes, lettuce, nitric oxide, autophagy

## Abstract

Lettuce (*Lactuca sativa* L.) contains various bioactive compounds that can reduce the severity of inflammatory diseases. This study aimed to identify therapeutic effects and underlying mechanisms of fermented lettuce extract (FLE) containing stable nitric oxide (NO) on collagen-induced arthritis (CIA) in mice and fibroblast-like synoviocytes (MH7A line) from patients with rheumatoid arthritis (RA). DBA/1 mice were immunized with bovine type II collagen and orally administered FLE for 14 days. On day 36, mouse sera and ankle joints were collected for serological and histological analysis, respectively. Consuming FLE inhibited RA development, suppressing pro-inflammatory cytokine productions, synovial inflammation, and cartilage degradation. The therapeutic effects of FLE in CIA mice were similar to those of methotrexate (MTX), which is typically used to treat RA. In vitro, FLE suppressed the transforming growth factor-β (TGF-β)/Smad signaling pathway in MH7A cells. We also demonstrated that FLE inhibited TGF-β-induced cell migration, suppressed MMP-2/9 expression, inhibited MH7A cell proliferation, and increased the expression of autophagy markers LC3B and p62 in a dose-dependent manner. Our data suggest that FLE could induce autophagosome formations in the early of stages of autophagy while inhibiting their degradation in the later stages. In conclusion, FLE is a potential therapeutic agent for RA.

## 1. Introduction

Rheumatoid arthritis (RA) is an autoimmune disease that mainly manifests as the chronic inflammation of joints. Its cause is a complex interaction between genetic, environmental and immunological factors [1]. Fibroblast-like synoviocytes (FLS) are located at the synovial intimal edge and play a critical role in RA pathogenesis. Activated FLS have similar characteristics, such as resistance to proliferation, invasion, and apoptosis of tumor cells, and they directly participate in synovial proliferation by migration to cartilage and bone to attach and invade [2]. Stimulation by inflammatory cytokines induces FLS proliferation and releases several factors, including inflammatory cytokines, chemokines, and matrix-degrading enzymes [3]. In addition, FLS proliferation interferes with immune cell regulation and leads to the destruction of the extracellular matrix, cartilage, and bone [4]. Therefore, it is important to modulate the biological behavior of FLS to ameliorate symptoms of RA. The MH7A cells are an FLS immortalized cell line transfected with the SV40 T antigen and are widely used to study the mechanism of RA. Transforming growth factor-β (TGF-β) is an important growth factor involved in the induction and proliferation of fibrosis associated with RA [5]. TGF-β has also been shown to be upregulated in FLS from RA patients in some studies [6]. TGF-β is a cytokine with diverse functions in proliferation, differentiation, angiogenesis, extracellular matrix production, and apoptosis. Activated TGF-β signaling phosphorylates and induces Smad, including pro-fibrotic Smad2/3 and anti-fibrotic Smad1/5/9 in progressive fibrosis [7]. Therefore, the TGF-β/Smad signaling pathway may contribute to RA-FLS.

The pathogenesis of autoimmune diseases, including RA, is associated with the dysregulation of programmed cell death [8]. Cell death involves degrading unnecessary or non-functional cellular components, and thus, it can be destructive or protective [9]. Several studies have demonstrated an association between cell death and RA in various cell types, including FLS. In RA, a decrease in FLS cell death leads to excessive synovial cell proliferation, promoting synovitis [10]. Therefore, novel therapeutic targets for RA should include pathways associated with cell death processes, including apoptosis, necroptosis, pyroptosis and autophagy. 

In particular, the correlation between RA pathogenesis and autophagy is entirely unknown. Autophagy is a degradation pathway that isolates and eliminates unnecessary cytoplasmic material such as damaged organelles and abnormal/non-functional proteins [11]. The target component is separated from other cellular components surrounded by an autophagosome and fused with a lysosome for decomposition [12]. Multiple proteins are involved in autophagy, with p62 and LC3 in particular being widely used to monitor autophagic flux, which is an indicator of overall autophagic degradation [13]. Cytoplasmic LC3 (LC3-I) conjugates with phosphatidylethanolamine to form LC3-II, which is recruited to the autophagosome membrane [14]. Autophagy targeting is completed when p62 binds to LC3-II, triggering lysosomal proteolysis and degrading p62 together with autophagosomes [15]. Disruptions in autophagy may be related to RA pathogenesis and may be targeted during therapy. 

Lettuce (family Asteraceae) is a globally consumed vegetable containing numerous vitamins, minerals, carotenoids, and polyphenols [16]. As a natural source of phytochemicals and bioactive nutritional compounds, lettuce has important health benefits, including cytoprotective and cholesterol lowering [17,18]. Moreover, some nutritional compounds in lettuce have been reported to protect hippocampal neurons against amyloid-α-mediated oxidative stress and apoptosis [19]. 

Fermentation improves the biological properties of raw materials by producing new bioactive compounds [20]. Some studies have shown that natural products such as fermented garlic, lettuce, and beans can improve menopausal symptoms and peripheral and central blood flow [21,22]. Additional studies have shown that fermented lettuce extracts (FLE) have an antidiabetic effect [23]. Thus, FLE has potential as an RA treatment; however, its application needs to be explored empirically.

In this study, we investigated the therapeutic efficacy of FLE, a naturally derived compound containing NO, stabilized for a long time. Using a collagen-induced arthritis (CIA) mouse model, we assessed whether RA prognosis improved after FLE consumption. We explored the effect or FLE on TGF-β and investigated the underlying mechanism of FLE-induced autophagy in MH7A cells.

## 2. Materials and Methods

### 2.1. Preparation of FLE

The study used FLE manufactured by food manufacturing company (HumanEnos LLC., Wanju, Republic of Korea) [21]. Ground lettuce was mixed with distilled water in 1:1 ratio, and then, approximately 1% (1.0 × 10^8^ cfu/mL) of generally recognized as safe (GRAS) grade microorganisms were added. Fermentation occurred at 30 °C for 21 days. Every factor that can affect fermentation was managed to maintain optimal conditions, including aeration, temperature, and pH. At the end of 21 days, the liquid product contained NO metabolites and antioxidants. The supernatant was then separated with a centrifuge (1.5 ton/Hr, disc separator, Alfatechkorea Corp., Seongnam, Republic of Korea) and then condensed with an evaporator (1.5 ton, Vacuum Evaporator, BDMPLANT, Gwangju, Republic of Korea), yielding brix = 4%. The condensed material was then frozen at –40 °C for 48 h and placed in a freeze dryer (1.5 ton, Vacuum Freeze Drier, Ajin E.S.R Co. Ltd., Daegu, Republic of Korea) for 72 h until reaching powder form. Nitrite levels in FLE were determined using Griess reagent (Promega, Madison, WI, USA), following manufacturer protocol (Table 1). The FLE used in this study was fermented for 21 days.

### 2.2. Animal Care

Male DBA/1J mice aged 7–8 weeks were obtained from Central Lab Animal Inc. (Seoul, Republic of Korea). The mice were housed in a specified pathogen-free animal facility under a 12 h light/dark cycle. They were fed ad libitum with a breeding diet (A03, SAFE, Rosenberg, Germany) containing 52.0% carbohydrates, 21.4% protein, 5.1% lipids, 4.0% fiber, 5.7% minerals, and 12.1% moisture. All experiments were approved by the Institutional Animal Care and Use Committee of Pusan National University, conducted in accordance with the National Institutes of Health Guidelines (PNU-2020-2757), and registered at preclinicaltrials.eu (PCTE0000355).

### 2.3. Generation of CIA Mice and Experimental Intervention 

The CIA tends to be more severe in male than in female mice [24,25]. The incidence of arthritis may reach 100% in males [26] and >80% in females in the CIA mouse model. The induction of CIA was as previously described [27]. Mice were immunized intradermally on day 0 via a tail-vein injection of 100 μg bovine type II collagen (CII; Chondrex inc., Redmond, WA, USA) emulsified with an equal volume of complete Freund’s adjuvant (CFA; Sigma-Aldrich, St. Louis, MO, USA). Immunization was boosted with an equal volume of CII emulsion and incomplete Freund’s adjuvant (IFA; Chondrex) on day 21. Normal mice were treated with Freund’s adjuvant only. After boosters, mice were randomly divided into four groups (*n* = 8): Normal, CIA, CIA + MTX, and CIA + FLE. Methotrexate (MTX) is a common RA drug, which is selected as the positive control. Oral administration is a common and cost-effective method of treatment. In addition, it is attractive because it is non-invasive, convenient, and enables long-time treatment [28]. Therefore, we accessed the oral administration of FLE to CIA mice. From days 22 to 35, FLE (75 μg nitrite/kg/day) was orally administered daily in the CIA + FLE group. Across the same period, MTX (1 mg/kg) was injected intraperitoneally once every 3 days for the CIA + MTX group. Normal mice were orally administered distilled water daily from day 22 to day 25 at the time of FLE administration. 

### 2.4. Clinical Assessment of Arthritis

Mice were closely monitored and scored three times a week after the primary collagen injection. Scoring was performed by three independent investigators for each limb on a four-point scale [27]: 0 = normal paw; 1 = erythema and mild swelling confined to the tarsal or ankle joint; 2 = erythema and mild swelling extending from the ankle to the tarsal; 3 = erythema and moderate swelling extending from the ankle to the metatarsal joints; 4 = erythema and severe swelling encompassing the ankle, foot and digits, or limb ankylosis. Final arthritis scores were obtained from summing scores of all four paws. Paw thickness was measured using an electric caliper placed across the ankle joint at its widest point. 

### 2.5. Enzyme-Linked Immunosorbent Assay (ELISA)

Mouse anti-CII antibodies (IgG, IgG1, and IgG2a; Chondrex) and mouse TNF-α, IL-1β/12, and IL-6 (R&D Systems, Minneapolis, MN, USA) were measured using ELISA kits according to the manufacturer’s instructions. Absorbance was read at 450 nm using a microplate reader (Tecan, Infinite M200, Austria).

### 2.6. IHC

On day 36, mice were euthanized to collect ankle joints. The samples were fixed in 10% phosphate-buffered formaldehyde solution for 24 h, then decalcified in a 10% ethylenediaminetetraacetic acid solution for 1 month, dehydrated and embedded in paraffin. TGF-β expression in ankle joints was measured as previously described [29]. Tissue sections (thickness, 3 μm) were incubated with primary antibodies against TGF-β (Abcam, Cambridge, MA, USA) overnight at 4 °C. Subsequently, they were incubated with HRP-conjugated goat anti-rabbit IgG antibody (DAKO, Carpinteria, CA, USA) to detect immunoactivity, which was followed by detection using a DAB solution kit (DAKO). The counterstain was hematoxylin. Stained specimens were visualized under a virtual microscope (Axio Scan.Z1; Carl Zeiss, Heidenheim, Germany). Histological results were the average of scores from three independent observers blinded to the experimental conditions. TGF-β expression was analyzed Image J (NIH; National Institutes of Health).

### 2.7. Histochemical Analysis

Tissue sections were prepared and stained with hematoxylin and eosin (H&E; Sigma-Aldrich) and safranin O (Sigma-Aldrich). Synovial inflammation and hyperplasia were determined based on the results of H&E staining [29]: 0, no signs of inflammation; 1, slight thickening of the lining layer or some infiltrating cells in the underlying layer; 2, slight thickening of the lining layer plus some infiltrating cells in the underlying layer; 3, thickening of the lining layer, cell influx in the underlying layer, and cell presence in the synovial space; 4, synovium highly infiltrated with many inflammatory cells. Cartilage damage was determined based on the results of safranin O staining: 0, no destruction; 1, minimal erosion limited to single spots; 2, slight-to-moderate erosion in a limited area; 3, more extensive erosion; and 4, general destruction. Stained specimens were visualized under a virtual microscope (Axio Scan.Z1; Carl Zeiss). Histology was assessed by three independent, blinded observers, and their scores were averaged for the final results.

### 2.8. Micro-Computed Tomography (CT)

Hind-paw images from mice in all four groups (*n* = 4) were acquired on day 36 using micro-CT (Quantum FX, Perkin Elmer, MA, USA) [30]. Ankle joints were scanned at a tube voltage of 90 kV, tube current of 160 μA, resolution of 20 μm scan time of 2 min. 

### 2.9. In Vivo Safety Evaluation

Preliminary safety tests for FLE were performed as previously described [31]. Healthy 6-week-old male C57BL/6 mice were randomly divided into two groups (*n* = 5–6), Control (*n* = 5) and FLE (*n* = 6). Each group received a single orally administered dose of FLE (75 μg nitrite/kg/day) or distilled water (control) daily for 14 days. Body weight was measured daily. Blood and tissue samples were collected 24 h after the final administration for hematologic and histochemical analyses. Levels of serum aspartate transaminase (AST), alanine transaminase (ALT), blood urea nitrogen (BUN), and creatinine were measured by GC Labs (Yongin, Republic of Korea) using the International Federation of Clinical Chemistry standard. Brain, lung, heart, liver, kidney, spleen, thymus, and testis tissues were fixed with 4% paraformaldehyde for 24 h and embedded in paraffin. Each sample was cut into 5 μm sections, processed for routine H&E staining, and visualized under a virtual microscope (Axio Scan.Z1; Carl Zeiss).

### 2.10. Cell Culture

Human synovial fibroblast cell line MH7A was obtained from the Prof. Sang-Il Lee (Gyeongsang National University, Jinju, Republic of Korea). The MH7A cells were cultured in Roswell Park Memorial Institute 1640 medium (RPMI 1640; Welgene, Gyeongsan, Republic of Korea) supplemented with 10% fetal bovine serum (FBS; Welgene) and penicillin/streptomycin (Welgene). Culture conditions were 37 °C in a humidified atmosphere with 5% CO_2_. Cells were treated with various FLE doses in the absence or presence of TGF-β (10 ng/mL; R&D Systems) or of chloroquine (CQ, 20 μM; Cayman, Ann Arbor, MI, USA) for 72 h. 

### 2.11. Cell Viability Assay

Cell viability was determined colorimetrically using 3-(4,5-dimethylthiazol-2-yl)-2,5-diphenyltetrazolium bromide (MTT; Duchefa, Haarlem, Netherlands) as previously described [32]. Cells were seeded in 96-well plates (1 × 10^4^ cells/well) and incubated for 24 h. After treatment with 62.5, 125, 250, and 500 μg nitrite/mL FLE for 24, 48, and 72 h, MTT (5 mg/mL) was added to each well, which was followed by a 2 h incubation. Supernatants were aspirated before 100 μL of DMSO was added to dissolve formazan crystals remaining in each well. Optical density per well was read at 570 nm using a microplate reader (Tecan).

### 2.12. Cell Proliferation Assay

Cell proliferation was assessed using the 5-bromo-2′-deoxyuridine (BrdU) cell proliferation ELISA kit (Abcam, Cambridge, MA, USA), as previously described [33]. Cells (1 × 10^4^) were seeded in a 96-well plate and treated with various doses of FLE for 72 h. BrdU was added to the appropriate wells, and cells were incubated for 4 h before being subjected to fixing solution. The plate was then washed and incubated with BrdU detector antibody for 1 h at room temperature. Peroxidase-conjugated secondary antibodies were added, which was followed by a 30 min incubation. Finally, cell proliferation was assessed using a spectrophotometer at 450 nm (Tecan).

### 2.13. Reverse-Transcriptase PCR (RT-PCR)

Total RNA was isolated from MH7A cells using Trizol reagent and synthesized into cDNA with the antiRivert Platinum cDNA synthesis master mix (GenDepot, Barker, TX, USA), following manufacturer protocol [31]. Target genes were amplified with RT-PCR using TOPsimple PCR DyeMix-Tenuto (Enzynomics, Daejeon, Republic of Korea) and the following primers: *ACTA1*, forward: 5′-GTGTTGCCCCTGAAGAGCAT-3′, reverse: 5′-GCTGGGACATTGAAAGTCTCA-3′; *COL1A*, forward: 5′-CCCGGGTTTCAGAGACAACTTC-3′, reverse: 5′-TCCACATGCTTTATTCCAGCAATC-3′; *MMP2*, forward 5′-AGCGAGTGGATGCCGCCTTTAA-3′, reverse: 5′-CATTCCAGGCATCTGCGATGAG-3′; *MMP9*, forward: 5′-GCCACTACTGTGCCTTTGAGTC-3′, reverse: 5′-CCCTCAGAGAATCGCCAGTACT-3′; *GAPDH*, forward: 5′-AAAATCAAGTGGGGCGATGC-3′, reverse: 5′-GATGACCCTTTTGGCTCCCC-3′. The cDNA was amplified by PCR with 25–30 cycles of denaturation at 95 °C for 30 s, annealing at 60 °C for 30 s, and elongation at 72 °C for 40 s. GAPDH was selected as the reference gene. The RT-PCR products (10 μL each) were resolved in a 1.5% agarose gel in Tris-acetic acid–EDTA (TAE) buffer and visualized with ethidium bromide under UV light. Relative expression of the target genes was quantified in Image J.

### 2.14. Western Blotting

Western blot analysis was performed as previously described [34]. MH7A cells were treated with IP lysis buffer (Thermo Fisher Scientific, Rockford, IL, USA) containing protease and phosphatase inhibitor cocktails (GenDEPOT) following manufacturer protocol. Lysates were centrifuged at 16,609× *g* (Hanil, Incheon, Republic of Korea) and 4 °C for 15 min. Protein concentrations were measured using at Bicinchoninic Acid Protein Assay Kit (Thermo Scientific). Samples were separated with sodium dodecyl sulfate-polyacrylamide gel electrophoresis (SDS-PAGE) and transferred to polyvinylidene difluoride (PVDF) membranes (Millipore, Darmstadt, Germany). Membranes were incubated overnight at 4 °C with primary antibodies phospho-Smad2/3 (1:1000, Cell Signaling, Danvers, MA, USA), phospho-Smad1/5/9 (1:1000, Cell Signaling), LC3B (1:1000; Cell Signaling), p62 (1:1000; Cell Signaling) and Beclin-1 (1:1000; Cell Signaling). Next, they were incubated with HRP-conjugated anti-rabbit (1:50,000; ENZO Life Science, Farmingdale, NY, USA) secondary antibodies for 1 h. An enhanced chemiluminescence kit (Amersham Pharmacia, Piscataway, NJ, USA) and ABI680 Analyzer (Amersham) were used to detect protein bands; signals were quantified in Image J. The loading control was β-actin antibody (1:5000; Sigma-Aldrich).

### 2.15. Cell Migration Assay

Cell migration was assessed using transwell chambers (8 μm pore size membrane; Cell Biolabs, San Diego, CA, USA), as previously described [35]. Cells were seeded in the upper chamber (1 × 10^5^ cells/well) with serum-free medium. To the lower chamber, medium containing 10% FBS was added, which was followed by FLE and recombinant human TGF-β (R&D Systems). After 24 h, cells found on the lower side of the transwell culture insert were considered migratory. These cells were fixed with 4% paraformaldehyde and stained with 0.1% crystal violet. Non-migratory cells (inside the membrane) were removed. Stained cells were photographed under a microscope (Nikon, Tokyo, Japan) and analyzed in ImageJ. The average number of migratory cells was calculated.

### 2.16. Detection of Autophagosome Formation

Autophagosome formation was analyzed using a CYTO-ID Autophagy Detection Kit (ENZO Life Science), which selectively labels accumulated autophagic vacuoles and autophagy flux with a green fluorescent dye [36]. Cells were cultured in 24-well plates with coverslips. Next, FLE, rapamycin (positive control), and CQ (negative control) were added, which was followed by incubation for 72 h. Hoechst and CYTO-ID detection reagents were dispensed into each sample for microscopic analysis. Images were obtained using confocal laser scanning (K1-Fluo; Nanoscope Systems, Daejeon, Republic of Korea).

### 2.17. Statistical Analysis

After checking for normality, one-way ANOVA/Bonferroni correction Kruskal–Wallis tests, or Mann–Whitney U tests were used to analyze between-group differences (OriginPro2020, OriginLab, Northampton, MA, USA). Significance was set at *p* < 0.05.

## 3. Results

### 3.1. FLE Treatment Reduced RA Severity in CIA Mice

**Figure 1:**
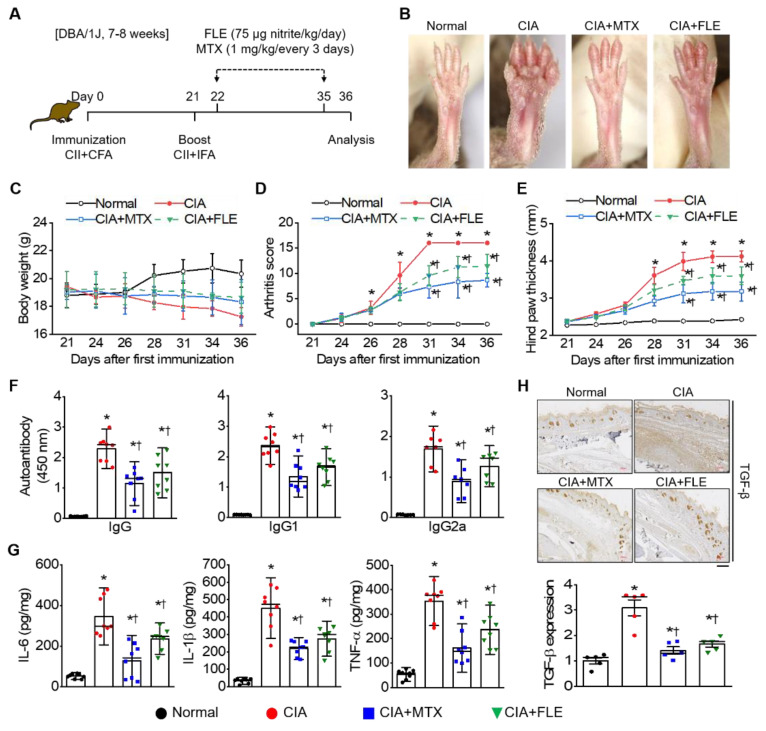
Fermented lettuce extract (FLE) attenuated RA symptoms in collagen-induced arthritis (CIA) mice. (**A**) Timeline of animal experiments. CIA DBA/1J mice were immunized with tail-vein injections of type II collagen. One group was orally administered FLE daily from days 22 to 35. Standard RA drug MTX was injected intraperitoneally once every 3 days as a positive control. (**B**) Representative photographs of hind paws, showing swelling and ankylosis in immunized mice. Body weight (**C**), arthritis score (**D**), and hind-paw thickness (**E**) were measured every 2 days during FLE administration. On day 36, serum levels of IgG, IgG1, IgG2a (**F**), and inflammatory cytokines IL-6, IL-1β, and TNF-α (**G**) were confirmed via ELISA. (**H**) Immunohistochemistry of TGF-β expression. Scale bar represents 200 μm. Data are shown as means ± SE (*n* = 8). * *p* < 0.05, compared to Normal. † *p* < 0.05, compared to CIA.

To investigate the effect of FLE on RA, we conducted an experiment as shown in Figure 1A. Histological observations in the hind paws of CIA mice revealed swelling and erythema. However, these symptoms decreased significantly in the CIA + FLE and CIA + MTX groups (Figure 1B). Regardless of MTX or FLE administration, mean body weight decreased in collagen-induced CIA mice (Figure 1C).

The CIA group showed a significant increase in the arthritis score when compared with the Normal group at day 36. This increase was reduced by 45.94% and 28.44%, respectively, by MTX and FLE treatment (*p* < 0.05; Figure 1D). Similarly, treatment with MTX and FLE showed reduced hind paw thickness by 28.82% and 12.62%, respectively, when compared to that of the CIA group (*p* < 0.05; Figure 1E). Next, we observed significantly higher serum levels of CII-specific IgG, IgG1, and IgG2a in CIA mice than in the normal group; MTX or FLE treatment attenuated this increase (*p* < 0.05; Figure 1F). The average absorbance value for each autoantibody of Normal, CIA, CIA + MTX, and CIA + FLE groups was IgG, 0.07 ± 0.00, 2.29 ± 0.15, 1.14 ± 0.17, and 1.50 ± 0.19; IgG1, 0.09 ± 0.00, 2.36 ± 0.15, 1.35 ± 0.16, and 1.66 ± 0.14; IgG2, 0.07 ± 0.01, 1.76 ± 0.08, 0.89 ± 0.13, and 1.31 ± 0.12. We then measured serum cytokine levels to determine how FLE affected inflammatory responses in CIA mice. ELISA results indicated that IL-6, IL-1β, and TNF-α levels were higher in CIA mice than in normal mice; the increase was attenuated by treatment with MTX and FLE (*p* < 0.05; Figure 1G). The average concentration for each pro-inflammatory cytokine of Normal, CIA, CIA + MTX, and CIA + FLE groups was IL-6 (pg/mg), 51.73 ± 4.09, 345.55 ± 33.12, 127.00 ± 29.75, and 236.15 ± 18.21; IL-1β (pg/mg), 33.29 ± 4.63, 450.88 ± 41.23, 219.07 ± 15.09, and 275.56 ± 23.88; TNF-α (pg/mg), 53.22 ± 6.77, 353.38 ± 23.72, 160.95 ± 23.36, and 235.52 ± 23.69. In the CIA group, the relative expression level of TGF-β in the synovial tissue was high when compared with that observed in the Normal group (Figure 1H). The expression level of TGF-β showed a significant decrease of 54.37% and 46.28% in the CIA + MTX and CIA + FLE groups, respectively (*p* < 0.05).

### 3.2. Administering FLE to CIA Mice Alleviated Histological Signs of RA

**Figure 2:**
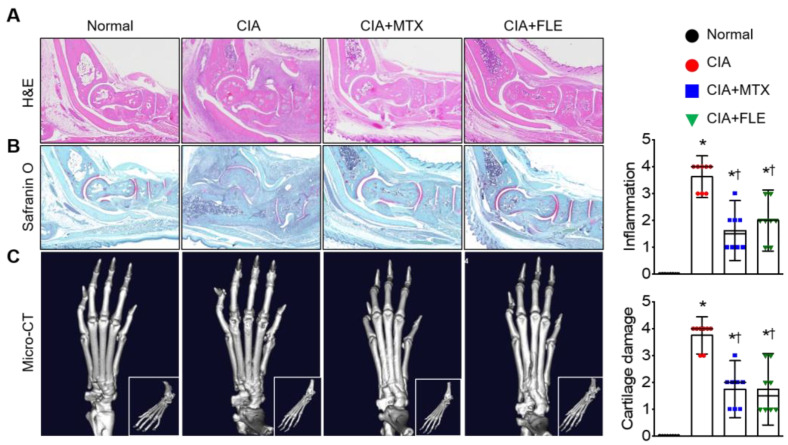
FLE alleviated synovial inflammation and joint destruction. Pathological features in mouse ankle joints were assessed. Representative photomicrographs of ankle joints stained with H&E (**A**) and safranin O (**B**). (**C**) Representative micro-CT images of ankle joints. Data are shown as means ± SE (*n* = 8). * *p* < 0.05, compared to Normal. † *p* < 0.05, compared to CIA.

Histological analysis of ankle joints and paws from CIA mice revealed characteristics of RA inflammation, such as edema, pannus formation, as well as cartilage and bone damage. These signs decreased significantly in the CIA + FLE and CIA + MTX groups (*n* = 8, *p* < 0.05; Figure 2A,B). Compared to the CIA mice, both MTX and FLE treatments significantly attenuated the synovial inflammation by 55.10 % and 48.21%, respectively. Similarly, the cartilage damage score showed a decrease of 53.33% and 50.00% in the CIA + MTX and CIA + FLE groups, respectively (*p* < 0.05). Pathological changes were confirmed using micro-CT, with CIA mice exhibiting more bone and cartilage degradation than normal mice (*p* < 0.05; Figure 2C). Furthermore, both MTX and FLE treatment significantly limited this degradation (*p* < 0.05).

### 3.3. Safety Assessment of FLE in Mice

**Figure 3:**
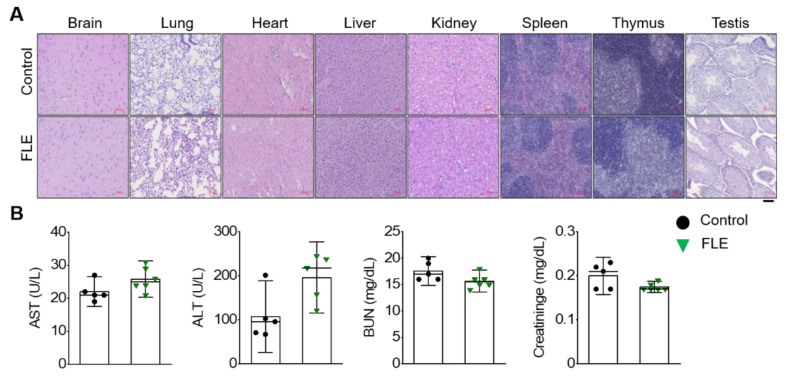
Safety profile of FLE. (**A**) Histological evaluation of H&E-stained brain, lung, heart, liver, kidney, spleen, thymus, and testis sections of healthy C57BL/6 mice after oral administration of 75 μg nitrite/kg/day FLE for 14 days. Scale bar represents 100 μm. (**B**) Serum levels of serum aspartate transaminase (AST), alanine transaminase (ALT), blood urea nitrogen (BUN), and creatinine after oral administration of FLE. Data are shown as means ± SE (Control, *n* = 5; FLE, *n* = 6).

To verify FLE safety, we performed a systemic toxicity test in healthy C57BL/6 mice after they consumed FLE. Histopathological observation of major tissues (brain, lung, heart, liver, kidney, spleen, thymus, and testis) confirmed the lack of abnormalities in either the control group or the FLE group (H&E staining, Figure 3A). In addition, serum AST, ALT, BUN, and creatinine levels did not differ between the two groups (*n* = 5−6, *p* < 0.05; Figure 3B). These results indicate that FLE does not cause systemic toxicity in mice.

### 3.4. FLE Attenuated TGF-β/Smad Mediated Migration in MH7A Cells

**Figure 4:**
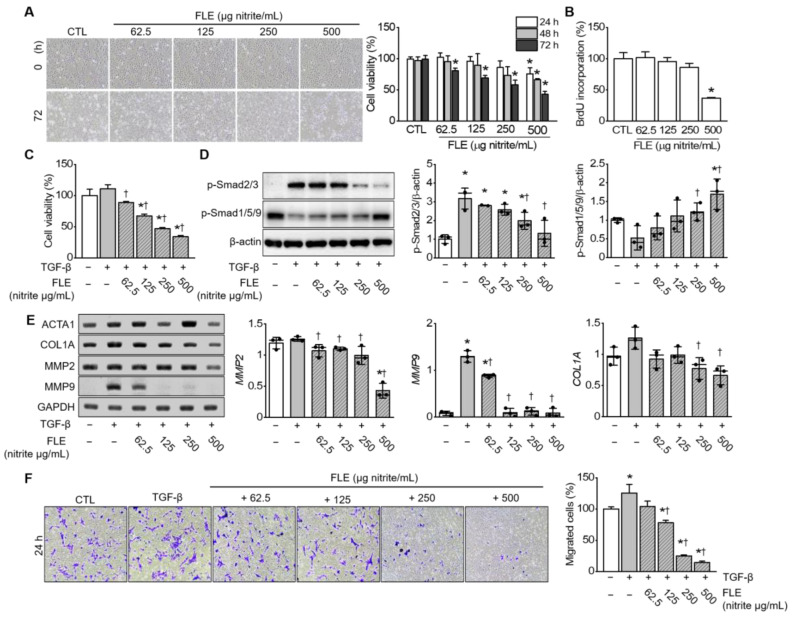
FLE suppresses the TGF-β/Smad signaling pathway in TGF-β-stimulated MH7A cells. (**A**) Representative morphological images of MH7A cells treated with FLE. Cells (*n* = 3) were exposed to various FLE concentrations (62.5, 125, 250, and 500 μg nitrite/mL) for 24, 48, and 72 h. Viability was measured using 3-(4, 5-dimethylthiazol-2-yl)-2, 5-diphenyltetrazolium bromide (MTT) assays. (**B**) 5-Bromo-2′-deoxyuridine (BrdU) was incorporated into MH7A cells after FLE treatment for 72 h (*n* = 3). (**C**) Effect of FLE and TGF-β (10 ng/mL) on MH7A cell viability at 72 h (*n* = 3). (**D**) Western blotting of phospho-Smad2/3 and phospho-Smad1/5/9 expression in MH7A cell lysates after 72 h treatment with TGF-β (*n* = 2−3). (**E**) RT-PCR detection of *ACTA1*, *COL1A*, *MMP2*, and *MMP9* mRNA in MH7A cells (*n* = 3). (**F**) Representative microscopic images of results from transwell migration assays. Data are shown as means ± SD (*n* = 3). * *p* < 0.05, compared to control (CTL). † *p* < 0.05, compared to TGF-β. The plus (+) sign represents a combination of FLE and TGF-β, as in +62.5, +125, +250, and +500. The minus (−) sign represents untreated samples.

Fibroblast-like synoviocytes (FLS) are important in RA pathogenesis because of their resistance to proliferation, migration, and apoptosis [37]. Regulating FLS biological behavior can improve RA symptoms. Here, we used the human FLS line MH7A to determine FLE cytotoxicity in vitro. The results of MTT assays indicated a significant decrease in cell viability at all FLE concentrations by 72 h (*n* = 3, *p* < 0.05; Figure 4A). Next, BrdU labeling revealed that 500 μg nitrite/mL of FLE significantly decreased MH7A proliferation by 72 h (*n* = 3, *p* < 0.05; Figure 4B).

We next stimulated MH7A cells with TGF-β (10 ng/mL) to determine the protective effects of FLE in an inflammatory environment. By 72 h, FLE had significantly suppressed cell viability in a dose-dependent manner (*n* = 3, *p* < 0.05; Figure 4C). TGF-β alone tended to increase cell viability but not significantly. Next, we investigated whether FLE is involved in the TGF-β-induced Smad signaling pathway. Cells were treated with different doses of FLE and 10 ng/mL TGF-β for 72 h. Next, Western blotting was used to determine phospho-Smad2/3 and phospho-Smad1/5/9 expression. After TGF-β stimulation, p-Smad2/3 and p-Smad1/5/9 expression increased and decreased, respectively, but FLE significantly reversed these changes (*n* = 2−3, *p* < 0.05; Figure 4D). To evaluate whether FLE could regulate TGF-β-induced fibrosis and the degradation of non-collagen matrix components in the joint, we used RT-PCR to determine *ACAT1*, *COL1A*, *MMP2*, and *MMP9* mRNA expression, and we found that *COL1A*, *MMP2*, and *MMP9* decreased significantly under FLE (*n* = 3, *p* < 0.05; Figure 4E). Consistent with these results, FLE also limited TGF-β-induced cell migration (*n* = 3, *p* < 0.05; Figure 4F).

### 3.5. FLE Induces Autophagosome Formation via Inhibiting Degradation in MH7A Cells

**Figure 5:**
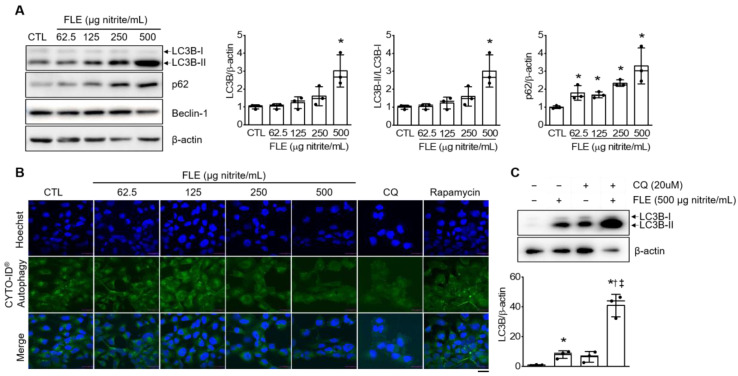
FLE induces autophagosome formation and thus regulates autophagy in MH7A cells. (**A**) Western blotting of LC3B, p62, and beclin-1 expression in MH7A cell lysates after 72 h treatment with FLE (*n* = 3). (**B**) Autophagy flux in MH7A cells after FLE treatment for 72 h. Cells were stained with CYTO-ID (green) and Hoechst (blue). Scale bar represents 30 μm. (**C**) LC3B expression in MH7A cell lysates after 72 h treatment with or without FLE and CQ. Data are shown as means ± SD. * *p* < 0.05, compared to CTL. † *p* < 0.05, compared to FLE. ‡ *p* < 0.05, compared to CQ. The plus (+) sign represents treated CQ or/and FLE. The minus (−) sign represents untreated samples.

We next investigated whether FLE regulates autophagy in MH7A cells. Western blotting indicated that FLE significantly upregulated autophagy markers LC3-II and p62 in a dose-dependent manner (*n* = 3, *p* < 0.05; Figure 5A). These results suggest that FLE induces autophagosome accumulation by suppressing their fusion with lysosomes. Treatment with FLE also did not alter Beclin-1 expression level, which is associated with autophagy induction. We next determined FLE involvement in the inhibition of autophagic flux. The CYTO-ID assay revealed a dose-dependent decrease in green fluorescence, reflecting reduced autophagy under FLE treatment (Figure 5B). Rapamycin-treated cells yielded a high green-fluorescence signal, whereas CQ-treated cells yielded a lower signal. We then measured LC3-II expression levels in MH7A cells after treatment with FLE and CQ. Because CQ blocks LC3-II degradation via inhibiting autophagosome-lysosomal fusion, we expect that combining CQ with FLE will have an additive effect on autophagic flux. Indeed, we observed elevated LC3-II expression under FLE treatment and even higher expression when CQ was also added (*n* = 3, *p* < 0.05; Figure 5C). Consequently, we suggest that FLE and CQ suppress late autophagy flux in a similar a manner.

## 4. Discussion

In our study, daily FLE treatment attenuated RA severity in CIA mice. FLE improved RA clinical symptoms, including hind-paw thickness and arthritis score. We also confirmed through histological analysis that the hind paws of FLE-treated mice experienced lower inflammation and cartilage damage. Furthermore, FLE consumption decreased the amount of IgG isotypes and pro-inflammatory cytokines, indicating that FLE can influence IgG-induced cytokine production in RA. Next, we performed in vivo tests using MH7A cells to elucidate the underlying mechanisms. The treatment of TGF-β-stimulated MH7A cells with FLE revealed that affected the TGF-β/Smad signaling pathway, decreasing COL1A1 and MMP-2/9 expression, and suppressed cell migration. Additionally, FLE inhibited MH7A survival and proliferation in a dose- and time-dependent manner. We then investigated how autophagy pathways were affected and found that FLE upregulated autophagy markers LC3-II and p62 but did not alter Beclin-1 expression. Moreover, FLE appeared to have a similar mechanism of action as CQ, which is an autophagy inhibitor.

Treatments for RA aim to relieve joint pain and inflammation, limit joint destruction or deformities, improve joint function, and induce sustained remission [38]. Nonsteroidal anti-inflammatory agents (NSAIDs), corticosteroids, and disease-modifying anti-rheumatoid drugs (DMARDs) are widely used drugs for RA [39]. Notably, DMARDs with high rheumatoid factor titers, such as methotrexate (MTX), sulfasalazine (SSZ), and hydroxychloroquine (HCQ), are used in the early stages of severe RA to reduce inflammation and prevent joint destruction [40]. However, these drugs have a variety of side effects, including hepatotoxic, pulmonary, and gastrointestinal problems [41]. In the effort to find treatments with fewer side effects, researchers have turned to natural food extracts that have a long history of medical applications [42,43]. Natural plant extracts used for RA typically act through several mechanisms, including anti-inflammatory activity, chondroprotection, angiogenesis inhibition, and antioxidant activity [44]. Advantages of natural plant extracts include greater customizability based on individual patient condition [45]. Moreover, many of the commonly used options are less toxic than existing drugs while containing bioactive compounds that affect signal transduction pathways relevant to diseases [46,47]. In this study, we selected lettuce as a natural medicine for RA because of previous reports regarding its anti-inflammatory, anti-diabetic, and antioxidant activity [17,18,23]. We specifically used FLE because the fermented product generates NO at a constant concentration for a long period of time. Treatment with FLE significantly limited hind-paw swelling, attenuated clinical symptoms, and inhibited IgG and pro-inflammatory cytokine expression in the serum of CIA mice. Additionally, FLE limited bone destruction, invasion, and inflammation. Overall, FLE significantly ameliorated RA symptoms.

In synovial tissue, FLS differentiate into myofibroblasts that produce and secrete extracellular matrix components, such as collagen. The transition to myofibroblasts drives increased proliferation, migration, and invasion [48]. The cytokine TGF-β plays a pivotal role in fibrosis and is upregulated in RA, with elevated expression in the synovial fluid and fibroblasts of patients. Specifically, TGF-β activates Smad phosphorylation in the Smad signaling pathway. The phosphorylation of Smad2/3 promotes fibrosis, whereas Smad1/5/9 exert anti-fibrotic effects [7]. Therefore, balancing Smad2/3 and Smad1/5/9 activation via TGF-β may be a viable strategy for RA treatment. Smad2/3 activation increases the expression of pro-fibrotic genes (e.g., collagen) and regulates the expression of matrix metalloproteases (MMPs) [48], which are major zinc-dependent endopeptidases that are involved in the invasion and degradation of the extracellular matrix. In RA synovial fibroblasts, increased MMP-2 and -9 production is especially associated with cartilage invasion [49]. Here, we investigated the effects of FLE on TGF-β-treated MH7A cells. We demonstrated that FLE affects TGF-β-induced Smad2/3 and Smad1/5/9 phosphorylation, downregulating COL1A, MMP-2, and MMP-9 expression to inhibit cell migration. Taken together, our data provide evidence that FLE relieves RA symptoms via the TGF-β/Smad signaling pathway.

An increase in RA-FLS causes synovial proliferation and is important in RA pathogenesis. Because of its resistance to cell death, activated RA-FLS exhibits tumor-like uncontrolled proliferation [4] that contributes to RA pathogenesis and progression [50]. In addition to resisting apoptosis, RA-FLS also appears capable of inducing autophagy. Studies have shown that autophagy increases in RA synovial tissues [51] and is implicated in protecting fibroblasts from cell death [52]. These findings underscore the importance of autophagy in controlling RA-FLS survival and proliferation. To investigate autophagy, we chose to measure LC3 and p62, two representative autophagy regulators. LC3 and p62 are essential proteins in autophagy, specifically autophagosome formation and fusion with lysosomes [53]. Here, we showed that the autophagy regulation of FLE inhibited MH7A proliferation and survival in a dose- and time-dependent manner. The dose-dependent increase in LC3B and p62 suggested that FLE regulates FLS proliferation and survival through inhibiting autophagosome–lysosomal fusion. In addition, our monitoring of autophagic flux and LC3B turnover revealed that FLE inhibited autophagy in a manner similar to CQ.

The main limitation of our study is that lettuce consists of various components, particularly after fermentation, and the influence of these components cannot be excluded. In addition, we have little data on whether RA is actually correlated with NO, which is the primary by-product of FLE. The exact reasons for the health benefits of fermented lettuce remain unclear, but some evidence suggests that NO production may play a role. Various studies have shown that the serum and synovial fluid of RA patients have high nitrite concentrations. Local inhibitors of nitric oxide (NO) synthesis could therefore be a therapeutic strategy for RA [54], but other studies have suggested that NO actually protects against inflammation. The release of pro-inflammatory mediators is regulated through local NO production, and in patients with RA, NO donors increase hyaluronic acid production by synovial cells [55,56]. Some evidence indicates NO does not mediate symptoms of late-stage RA (e.g., chronic inflammation and joint destruction) nor is its production fundamentally associated with arthritis susceptibility or severity [57]. Therefore, experiments directly introducing NO could help address these apparent contradictions. Whether TGF-β contributes to RA progression also remains controversial. We further note that a recent study on autophagy in RA contradicted our findings, suggesting that autophagy may have a dual role in RA-FLS survival [58]. To clarify the effect of FLE on RA, future research should investigate the molecular properties of FLE components and identify the signaling pathways involved in autophagy.

Despite these limitations, our findings in a mouse model suggest that FLE ameliorates severe clinical symptoms of RA. Moreover, we confirmed that FLE regulates fibrosis and cell migration through the TGF-β/Smad signaling pathway and inhibits RA-FLS proliferation and survival via downregulating autophagy. Thus, FLE administration has strong potential for use as an RA treatment

## 5. Conclusions

Collectively, our results demonstrate the effects of FLE on an RA mouse model and the human fibroblast synoviocyte line MH7A. Treatment of CIA mice with FLE had a similar effect as a standard RA drug (MTX), attenuating symptom severity. Molecular analyses suggest that the mechanism underlying FLE efficacy is the inhibition of cell migration and proliferation through the TGF-β/Smad signaling pathway and autophagy. We conclude that FLE may be a promising addition to RA therapy.

## Figures and Tables

**Table 1:** Nitrite contents of fermented lettuce extract.

Fermentation Period (Day)	0	2	21
Nitrite (μg/mL)	N.D *	3.34 (3.28–3.41) **	1127.41 (1113.32–1141.50) **

* N.D: not detected. **: 95% confidence intervals (CIs).

## Data Availability

The study did not report any data.
